# Challenges in the Diagnosis and Treatment of Aneurysmal Bone Cyst in Patients with Unusual Features

**DOI:** 10.1155/2019/2905671

**Published:** 2019-08-04

**Authors:** Ziyad M. Mohaidat, Salah R. Al-gharaibeh, Osama N. Aljararhih, Murad T. Nusairat, Ali A. Al-omari

**Affiliations:** ^1^Orthopedic & Spine Surgeon, King Abdullah University Hospital, Irbid 22110, Jordan; ^2^Assistant Professor, Orthopedic Surgery Division, Special Surgery Department, Faculty of Medicine, Jordan University of Science and Technology, Irbid 22110, Jordan; ^3^Orthopedic Surgery Resident, King Abdullah University Hospital, Jordan University of Science and Technology, Irbid 22110, Jordan

## Abstract

**Objectives:**

Aneurysmal bone cyst (ABC) is a benign but locally aggressive tumor. It has several challenging features. The aim of this study is to identify challenges in the diagnosis and treatment of ABC especially in patients with unusual features.

**Methods:**

This retrospective study involved medical record review of primary ABC patients with one or more of the following features: unusual clinical presentation with a mass or a pathological fracture especially at an unusual age, rare locations, radiological findings suggesting other diagnoses especially sarcoma, and a nondiagnostic histopathology of biopsy samples.

**Results:**

25 patients (17 males and 8 females) were included. Most patients were either younger than 10 or older than 20 years. 10 patients presented with a mass or a pathological fracture. Unusual locations include the scapula, the olecranon, the hamate, the calcaneus, and the first metatarsal bone. Extension into the epiphysis occurred in 2 patients with proximal fibula and olecranon ABCs. Two separate synchronous cysts existed in the proximal epiphysis and middiaphysis of one humerus. Radiological imaging suggested other primary diagnoses in 8 patients. Core needle biopsy was diagnostic in only 2 of 7 patients. The main treatment was intralesional resection/curettage with bone grafting. Wide resection was performed in 4 patients. Recurrence rate was 28%. Recurrence risk factors included the following: age less than 10 years, male gender, and proximal femur location. Late recurrence occurred in 3/7 patients. One patient with asymptomatic radiological recurrence showed subsequent spontaneous resolution one year later.

**Conclusions:**

This study presented multiple unusual features of ABC including: unusual age, rare locations, and nondiagnostic radiological and histopathological findings. These features can complicate the diagnosis and management. Given these features, especially with pathological fractures, a well-planned incision, the use of frozen section examination, and the application of either external fixation or plate osteosynthesis for fracture fixation can be recommended.

## 1. Introduction

Cystic lesion of the bone is one of the common challenges that might be encountered by orthopedic surgeons. Aneurysmal bone cyst (ABC) is a major differential diagnosis in such lesions. Although first described in 1942 by Jaffe and Leichstein [1, 2], the true etiology is still unknown [3].

ABC is a benign expansile osteolytic lesion that typically affects the metaphysis of long bones in young patients during their second decade of life [4–8]. Although benign, it can be locally aggressive. The radiological features can mimic both benign and malignant tumors [2].

ABC can be either primary with no preexisting lesions or secondary to other underlying pathologies [9]. This adds to the challenge in ABC diagnosis since it may be confused even after histopathological examination with more serious diagnosis especially telangiectatic osteosarcoma [10]. Cytogenetic studies can be used to confirm the diagnosis of primary ABC since specific translocations of the ubiquitin-specific protease (USP) 6 gene has been identified only in primary ABCs [4, 11, 12].

Several ABC treatment modalities had been utilized including wide resection [2, 13–15], intralesional resection/curettage with or without different adjuvants [6, 15–19], radiation [20], curopsy [21], embolization [22–24], intralesional sclerotherapy [25, 26], and more recently denosumab [27]. These methods have been reported with variable risk of recurrence, a major concern when treating ABC.

In this cohort, we present a group of patients who had one or more of the unusual features of ABC. In addition, the diagnostic and management considerations of these patients with such unusual features are presented.

## 2. Patients and Methods

This retrospective study involved review of the medical records of patients with primary ABC diagnosis who were treated at King Abdullah University hospital from January 2009 to June 2018. Patients included in this cohort have one or more of the following features: the less common clinical presentation with a palpable mass or with a pathological fracture especially at an unusual age, the cyst presented at rare locations, the radiological findings suggestive of other differential diagnoses especially sarcoma, and the histopathological examination, after either open or needle biopsy, suggested other diagnoses or could not exclude an underlying more serious pathology.

The management approach, outcome, and follow-up were reviewed for patients considered to have one or more of these unusual ABC features. This study was approved by the University Research Committee.

## 3. Results

A total of 25 patients were included as having one or more of the unusual features of ABC. There were 17 males and 8 females. Their average age at the time of diagnosis was 12.8 years (3 to 32 years). 12 patients were younger than 10 years, among which 7 patients were 5 or less years of age. While 7 patients presented during the second decade, 6 patients were older than 20 years.

**Figure 1:**
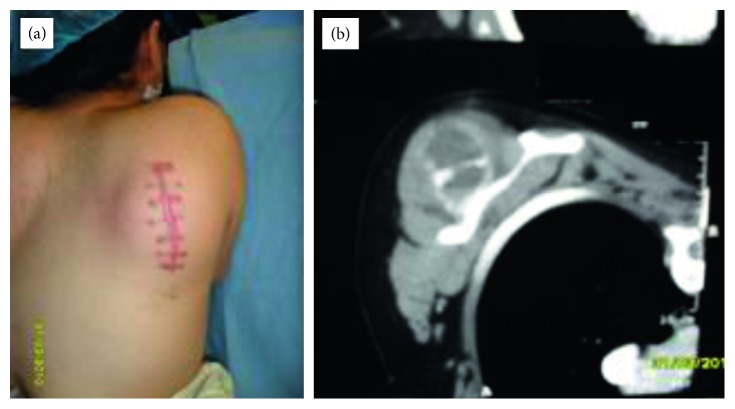
(a) Posterior Scapular mass with broad scar after resection attempt. (b) Axial CT showing soft tissue mass extending posterior to the scapula.

**Figure 2:**
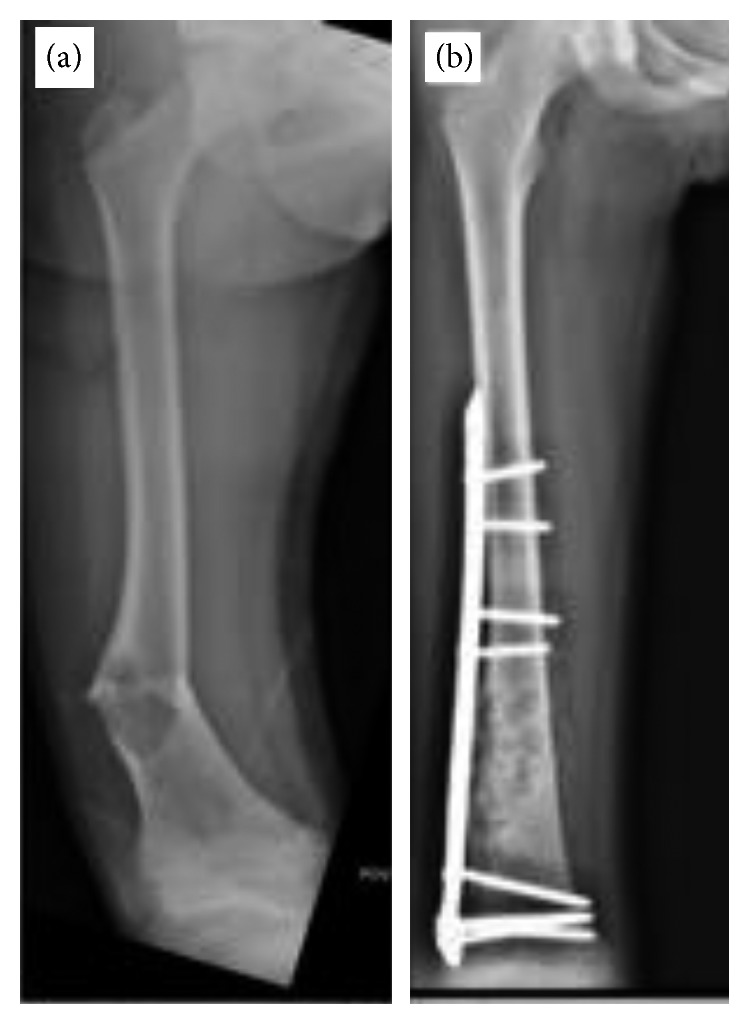
(a) AP X-ray with pathological fracture through a diaphyseal ABC. (b) AP X-ray after intralesional resection/curettage followed by fracture fixation with plate and screws.

Regarding clinical presentation, most patients (15/25) presented with localized pain. Four patients had a palpable mass as their initial complaint (Figure 1(a)). While three patients had their initial presentation with a pathological fracture (Figure 2(a)), another three patients were referred with a pathological fracture at the time of recurrence.

**Figure 3:**
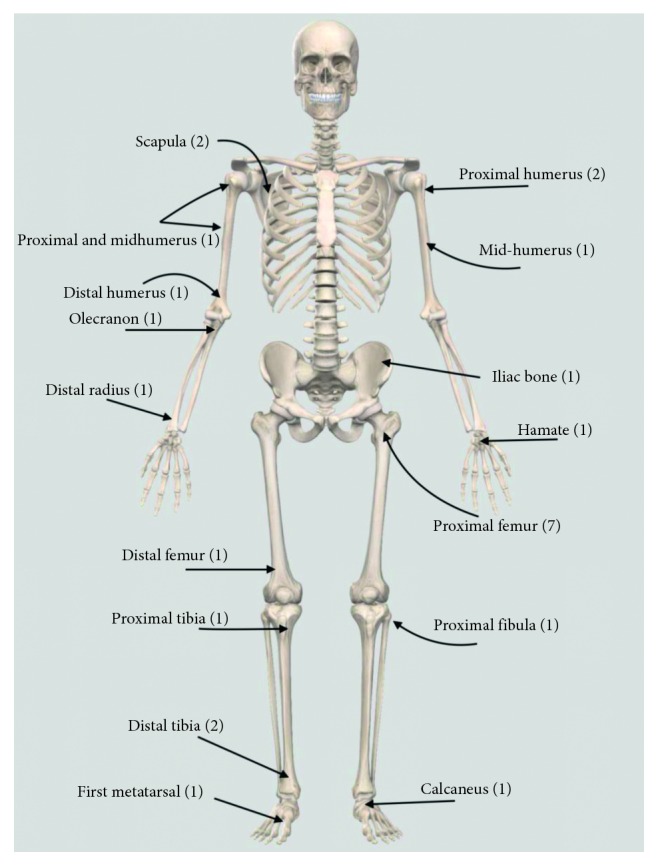
Anatomical distribution of aneurysmal bone cysts.

**Figure 4:**
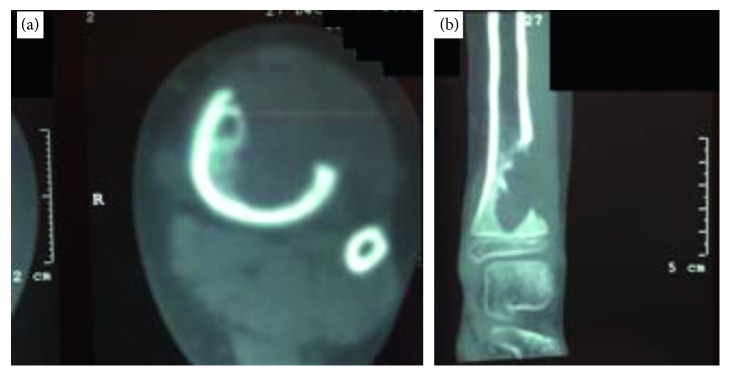
(a) Axial CT showing destruction of the anterior tibial cortex with extension into soft tissue. (b) Coronal CT showing ill-defined distal tibial lesion with cortical destruction.

**Figure 5:**
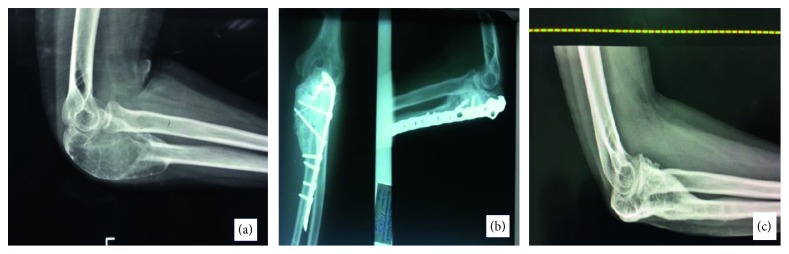
(a) Lateral elbow X-ray showing expansile cystic lesion involving the whole olecranon. (b) AP and lateral elbow X-rays after surgery with olecranon anatomical locking plate. (c) Lateral X-ray 4 years after removal of metal showing complete healing of the lesion.

**Figure 6:**
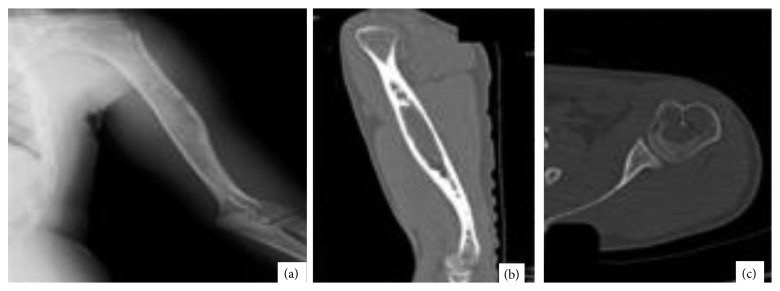
(a) AP X-ray of the humerus showing cystic lesions involving the proximal and midhumerus. (b, c) Sagittal and axial CT for the same patient showing expansile cysts of the mid and proximal humerus, respectively.

The anatomical location of the reported cysts included long and flat bones (Figure 3). The involved flat bones were the scapula, the hamate, the calcaneus, and the iliac bones. Although confined within the cortices of the hamate and calcaneus, the cysts involving the iliac bone and the scapulae had a periosteal location (Figure 1(b)). Within long bones, about half of the patients (13/25) had the cyst confined to the metaphysis. However, in one patient with distal tibial metaphyseal ABC (Figure 4), the cyst had extracortical extension into the surrounding soft tissue. The cyst was located in the diaphysis in two patients with humerus and distal femur ABCs (Figure 2(a)). Epiphyseal extension occurred in two patients with fibular and olecranon ABCs (Figures 5(a) and 5(b)). In one patient with humerus ABC, two synchronous separate cysts existed in the proximal epiphysis and middiaphysis (Figure 6).

Radiological workup included primarily X-ray imaging. CT, MRI, and bone scan were done selectively. The radiological evaluation suggested ABC as the primary differential diagnosis in 17/25 patients. Sarcoma was the primary radiological diagnosis in two patients (Figures 1(b) and 4). Giant cell tumor of bone was the primary differential diagnosis in three patients with proximal fibula, iliac bone, and one scapular cyst. In 3 patients with proximal femur cyst; fibrous dysplasia was suggested in one cyst while unicameral bone cyst was the main radiological diagnosis in the other two cysts.

**Figure 7:**
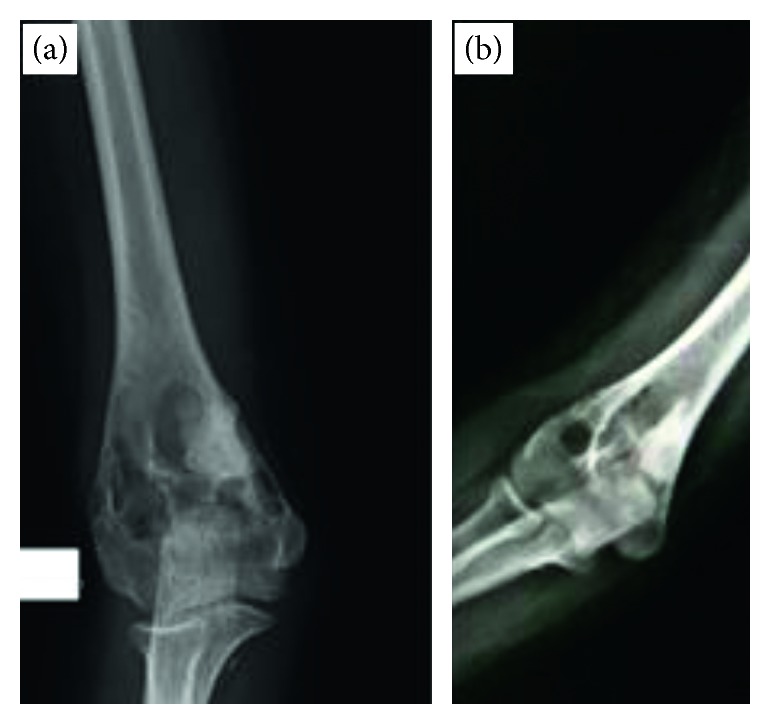
(a) AP X-ray of the distal humerus solid variant ABC about 4 years after surgery showing signs of recurrence. (b) X-ray repeated one year later with no further intervention showing almost complete healing and remodeling of the cyst.

**Figure 8:**
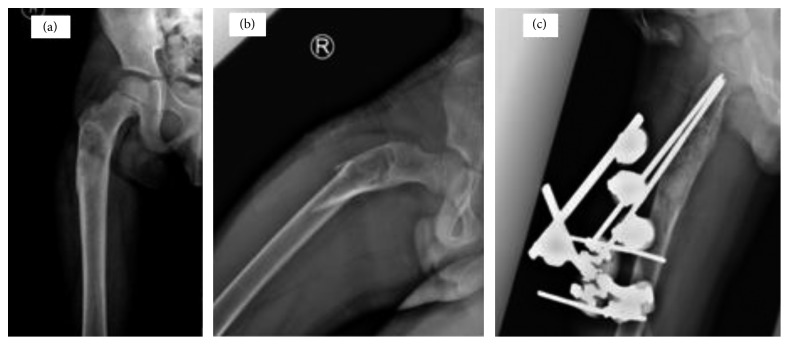
(a) AP Femur X-ray with proximal femur cyst diagnosed as benign fibrous histiocytoma. (b) The patient presented 2 years later with a pathological fracture. Final pathology revealed ABC. (c) Lateral X-ray of proximal femur with external fixator showing partial healing of the cyst and a well-maintained fracture reduction about 6 weeks after surgery.

All patients in this study had a final pathological diagnosis of primary ABC among which two patients with distal humerus (Figure 7(a)) and distal tibia cysts (Figure 4) showed the histologic solid variant of ABC. In 15 patients, no biopsy prior to definitive surgery was obtained. However, frozen section examination was used in 6 patients before proceeding with further intralesional resection/curettage. Among these 15 patients, one patient, with proximal femur cyst (Figure 8(a)), was diagnosed with benign fibrous histiocytoma. However, 2 years later, he presented with a pathological fracture due to recurrence (Figure 8(b)). Histopathology at this time confirmed the diagnosis of ABC.

Biopsy methods included open and core needle biopsy in 3 and 7 patients, respectively. While open biopsy confirmed ABC diagnosis in 2 patients, a more serious pathology could not be excluded in the third patient. Core needle biopsy provided the diagnosis in 2 patients while it was inadequate in 2 other patients. In the remaining 3 patients, the diagnoses were unicameral bone cyst, nonossifying fibroma, and giant cell tumor of bone.

As for treatment, wide resection was performed in 4 patients. Intralesional resection/curettage with bone grafting was done in the rest of the patients. No adjuvants were added after curettage in 10 patients. Different adjuvants including bone cement, liquid nitrogen, and hydrogen peroxide were used in 1, 3, and 7 patients, respectively. Different bone graft options including demineralized bone matrix (DBM), allograft chips, autograft, and tricalcium phosphate were used either alone or in combination. Different orthopedic hardware options were used including plates (Figure 2(b)), external fixators (Figure 8(c)), and intramedullary nails.

The follow-up period of these patients after treatment ranged from 12 months to more than 120 months with an average of 55.2 months. While none of the patients who had wide resection as their treatment developed a recurrence, 7 of the 21 patients who were treated with intralesional resection/curettage and bone grafting developed at least one recurrence (Table 1). Their first recurrence occurred at an average of 29 months after surgery. Three patients presented with a pathological fracture at the time of recurrence. Subsequent management of the recurrent ABCs involved mainly recurettage with bone grafting. Elastic intramedullary nails, plate with screws, and external fixators were used to fix the concurrent pathological fractures. However, the distal humerus ABC received no treatment since the patient was asymptomatic with almost complete healing of the cyst on radiographs repeated one year later (Figure 7(b)).

## 4. Discussion

This study investigates the different unusual clinical, radiological, and histopathological features of ABC that can reflect significantly on the management approach.

In this study, most patients (18/25) were either younger than 10 years of age or older than 20 years. This age can represent an unusual age since the peak incidence of ABC occurs in the second decade of life [7, 8, 23, 28, 29]. ABC might not be included in the initial differential diagnosis especially in older patients given that more than 90% of ABC occurs before the age of 20 years [30, 31]. In addition, the younger age can complicate the management with increased risk of recurrence [32].

ABC most commonly presents with a localized pain [13, 31, 33]. However, the less common presentation as a palpable mass or with a pathological fracture can further complicate the diagnosis and treatment [2, 6, 13, 23, 31, 34, 35]. Pathological fracture as an initial ABC presentation, as in 3 patients in this series, has been reported with variable rates and controversial management approaches by different series [2, 6, 14, 31, 34–39]. In addition, ABC presentation with a palpable mass especially at an older age or unusual location for ABC may be confused with more common pathologies. For instance, one patient with a scapular ABC (Figure 1(a)) was referred after an attempted excisional biopsy of what was thought to be a possible lipoma overlying the scapula.

The unusual ABC locations in this study include the scapula, the olecranon, the hamate, the calcaneus, and the first metatarsal bone [2, 8, 40–42]. In these locations, ABC diagnosis might not be considered. ABC typically affects the metaphysis of long bones [9]. Hence, when a cyst is located in the diaphysis or extending into the epiphysis, other diagnoses can be considered more typical. Also, as was observed in one patient, when two separate synchronous cysts exist in a single bone (Figure 6), ABC diagnosis can be excluded since multicentric ABC is extremely rare [43, 44].

ABC has a variable radiological appearance with no pathognomonic radiological features [2, 4, 45]. Although in most patients (17/25), the radiological diagnosis was concordant with the final histopathological diagnosis of ABC, other diagnoses were suggested in the remaining patients. This can complicate the radiological and histopathological correlation especially when sarcoma is suggested due to cortical destruction or soft tissue extension (Figures 1(b) and 4). In such situations, cytogenetic studies, although not performed in this study, can be suggested to confirm the diagnosis of primary rather than secondary ABC.

The role of image guided needle biopsy prior to the definitive surgery is debated [2, 4]. Core needle biopsies done in 7 patients were diagnostic in only 2 patients. This might be explained by the fact that the tissue obtained might not be adequate or representative of the whole lesion. Although frozen section histopathology did not provide the definitive diagnosis in patients included in this cohort, it was helpful to confirm the benign nature of the lesion. Therefore, it can be a reasonable step prior to proceeding with more definitive surgery.

The optimal treatment of ABC is controversial [13, 33], which can be related to different experiences of the treating physicians in different centers (Table 2). Due to the continuously evolving treatment options of ABC, selective arterial embolization as a less invasive treatment modality can be considered a suitable initial option especially with the encouraging results of the more recent large series of ABC treatment with embolization [22, 23]. Similar to other several studies [2, 4, 6, 8, 15, 19, 46–48], the main treatment of ABC in this series involved intralesional resection/curettage with bone graft. However, the unusual locations added to the therapeutic challenge. In the distal humerus ABC, the anatomy of the distal humeral metaphysis made it difficult to adequately curette cyst walls especially using single anteromedial approach. Also, in the patient with the double humerus ABCs, the cysts were approached by two separate bone windows through deltopectoral approach. Olecranon ABC (Figure 5(a)), with almost complete involvement of the articular surface, did add to the management challenge since failure to preserve the joint can be associated with significant morbidity. Adding tricortical iliac crest graft held with a locking plate (Figure 5(b)) was helpful to support the articular surface and preserve the joint (Figure 5(c))

Wide resection of ABC can be associated with significant morbidity. However, it can be indicated especially in expandable locations in which significant functional deficit is not expected after resection [4, 49, 50]. In this study, wide excision was performed for ABCs involving the scapula, the iliac bone, and the proximal fibular epiphysis. Scapular ABC patients presented at an older age for ABC. In addition, their MRI scans showed heterogenous soft tissue mass which was also demonstrated in the iliac bone ABC. Needle biopsy of the ABC involving the proximal fibular epiphysis suggested giant cell tumor of bone rather than ABC. Given these features together with the fact that ABC can be associated with an underlying sarcoma, wide excision in such expandable locations can be the treatment of choice.

As for pathological fractures treatment, several authors had suggested that a formal histopathological diagnosis should be obtained prior to further interventions [36–38]. Since a more serious pathology may not be excluded at least initially even after an open or a needle biopsy of a suspected ABC, our treatment included a planned surgical approach to avoid further tissue planes contamination. This can make wide resection still possible should the final pathology show an underlying primary sarcoma. In addition, we used frozen section examination before proceeding with further definitive intralesional resection/curettage. Also, we used either external fixation (Figure 8(c)) or plate osteosynthesis (Figure 2(b)) rather than intramedullary devices for fracture fixation so as to minimize further contamination of the medullary canal.

Recurrence rates after different therapeutic approaches vary widely, ranging from 0% to more than 59% [2, 4, 8, 15, 19, 29, 35, 49–52]. Recurrence rate in this study was 28%. In this study, most patients with recurrence were males and were less than 10 years of age. The proximal femur was the most frequent location involved while ABC in the unusual locations including the scapula, the olecranon, the hamate, the calcaneus, and the first metatarsal bone showed no recurrence during follow-up. These risk factors are similar to what is reported by other studies [2, 32]. It is worth mentioning that none of the patients who had wide resection had a recurrence. However, one third of patients treated with intralesional resection/curettage and bone graft (Table 1) developed a recurrence. Based on these findings, it may be recommended that whenever possible, wide resection be considered as the first option. Also, a closer follow-up can be recommended when treating a young male with ABC especially in the proximal femur. Additionally, a pathological fracture at the site of a previous treated ABC can be considered a sign of recurrence.

The rare atypical solid variant of ABC has been reported by several authors to have a better prognosis with lower recurrence risk compared to the usual ABC [31, 53–55]. However, Johnson and Caracciolo [56] reported increased recurrence risk of the solid ABC variant. In this study, both solid ABC patients developed a recurrence (Table 1). This can be explained by the inadequate curettage especially in the distal humerus ABC as already mentioned. In addition, both patients were younger than 10 years of age.

Late recurrence, more than 3 years after treatment (Table 1), was observed in 3 patients. This can represent an unusual feature given that most recurrences occur during the first 2 years after treatment [6, 33]. Long-term follow-up might be warranted given the possibility of late recurrence. Interestingly, the distal humerus ABC patient showed an unusual asymptomatic radiological recurrence more than 4 years after surgery which resolved one year later with no further treatment (Figure 7). This might represent the atypical ABC behavior of spontaneous resolution [57].

## 5. Conclusions

This study presented multiple unusual features of ABC including unusual age, rare locations, and nondiagnostic radiological and histopathological findings. These features can complicate both the diagnosis and management of ABC. Given these features, especially with pathological fractures, a well-planned incision, the use of frozen section examination, and the application of either external fixation or plate osteosynthesis for fracture fixation can be recommended. Recurrence is common especially in young males with proximal femur cyst. Closer follow-up can be warranted.

## Figures and Tables

**Table 1:** Clinical and management features of the 7 recurrent ABC patients.

Location	Age (years)	Sex	Adjuvant	Bone graft	Time to first recurrence (months)
Distal Humerus^S^	8	F	NO	Chips, Vitoss	48
Proximal Humerus	4	M	H_2_O_2_	Chips	24
Distal Tibia^S^	8	M	LN	Chips	6
Proximal Femur^P^	13	M	Cement	BC	55
Proximal Femur^P^	3	M	No	DBM	24
Proximal Femur	5	M	LN	DBM, chips	12
Proximal Humerus^P^	8	M	No	DBM	37

M: male; F: female; H_2_O_2_: hydrogen peroxide; LN: liquid nitrogen; BC: bone cement; DBM: demineralized bone matrix; chips: allograft chips; Vitoss: calcium triphosphate bone graft substitute. ^S^Solid ABC variant. ^P^Pathological fracture.

**Table 2:** ABC treatment modalities and their recurrence rates reported by different series.

Authors	Year	Main treatment	Patients (*N*)	Recurrence rate (%)
Vergel De dios et al. [6]	1992	Curettage and bone graft	124	21.8
Marcove et al. [15]	1995	Curettage and cryosurgery	51	17.6
Marcove et al. [15]	1995	Curettage and bone graft	44	59
Mankin et al. [2]	2005	Curettage and allograft	101	21
Rastogi et al. [25]	2006	Sclerotherapy	72	2.7
Varshney et al. [26]	2010	Sclerotherapy	45	6.7
Rossi et al. [23]	2010	Selective arterial embolization	36	39
Steffner et al. [16]	2011	Curettage, burring, and argon beam coagulation	40	7.5
Reddy et al. [21]	2014	Curopsy	102	18.6
Erol et al. [14]	2015	Curettage, burring, and graft	59	7
Terzi et al. [24]	2017	Selective arterial embolization	23	26
Rossi et al. [22]	2017	Selective arterial embolization	88	18.2
Zhu et al. [20]	2017	Radiation	12	0
Palmerini et al. [27]	2018	Denosumab	9	22.2
Aiba et al. [17]	2018	Endoscopic curettage	30	10
Syvänen et al. [18]	2018	Curettage and bioactive glass	18	11

## Data Availability

The data used to support the findings of this study are available from the corresponding author upon request.
